# The Comparison of the Initial TIMI Flow Grade in Acute ST-Elevation Myocardial Infarction Patients Receiving Ticagrelor vs. Clopidogrel before Undergoing Primary Percutaneous Coronary Intervention: A Prospective Cohort Study

**DOI:** 10.1155/2024/6632656

**Published:** 2024-02-05

**Authors:** Amin Elahifar, Ali Rafati, Mohammad Javad Alemzadeh-Ansari, Yeganeh Pasebani, Behshad Naghshtabrizi, Younes Mohammadi, Seyed Kianoosh Hosseini

**Affiliations:** ^1^Rajaie Cardiovascular Medical and Research Center, Medical School, Iran University of Medical Sciences, Tehran, Iran; ^2^Cardiovascular Intervention Research Center, Rajaie Cardiovascular Medical and Research Center, Iran University of Medical Sciences, Tehran, Iran; ^3^Farshchian Heart Center, Hamadan University of Medical Sciences, Hamadan, Iran; ^4^Department of Epidemiology, School of Public Health, Hamadan University of Medical Sciences, Hamadan, Iran

## Abstract

**Objective:**

Primary percutaneous coronary intervention (PCI) is the best treatment for acute ST-elevation myocardial infarction (STEMI). Evidence is in favor of ticagrelor over clopidegrel in STEMI patients regarding the reduction of stent thrombosis risk during and after PCI. We compared initial thrombolysis in myocardial infarction (TIMI) flow in STEMI patients on ticagrelor vs. clopidogrel.

**Methods:**

This prospective cohort recruited 160 patients with acute STEMI, referred to the emergency department of Farshchian Heart Center, during March 2018–2019. Before angiography, the patients received clopidogrel (600 mg) or ticagrelor (180 mg) on top of aspirin. Initial TIMI flow was compared between the two groups as the primary outcome. A logistic regression was performed to calculate the predictors of initial TIMI flow. Analyses were performed using R, version 4.2.1.

**Results:**

In ticagrelor and clopidogrel groups, the mean ± standard deviation age of the patients was 59.46 ± 13.11 and 61.34 ± 11.08 years (*p* value = 0.33), respectively. In the ticagrelor and clopidogrel groups, initial TIMI flow grades were as follows: 0 : 50% and 71.2%, I: 26.2% and 16.2%, II: 12.5% and 10%, and III: 12.9% and 2.5%, respectively (*p* value = 0.005). Final TIMI flow grades were as follows: I: 26.2% and 16.2%, II: 7.5% and 13.8%, and III: 66.3% and 70%, respectively (*p* value = 0.41). Ticagrelor was associated with significantly higher initial TIMI flow grade compared to the clopidogrel group (adjusted odds ratio: 2.90 (95% CI: 1.51–5.72)).

**Conclusion:**

In STEMI patients who were candidates for primary PCI, ticagrelor administration led to a better initial TIMI flow grade compared to clopidogrel.

## 1. Introduction

Acute ST-segment elevation myocardial infarction (STEMI) is one of the most important causes of mortality around the globe. The disease occurs following acute thrombotic occlusion of an epicardial coronary artery. Coronary artery occlusion usually occurs due to atherosclerotic plaque rupture and thrombosis [1]. Timely reperfusion through primary percutaneous coronary intervention (PPCI) is the most important treatment to improve the prognosis of patients with STEMI [1, 2].

Platelet activation, followed by aggregation, plays a key role in the process of STEMI. Administration of platelet P2Y12 inhibitors on top of aspirin before PPCI is considered an important therapeutic strategy that helps restores the perfusion to the ischemic area and prevents stent thrombosis [1–3].

Clopidogrel is an oral inhibitor of the platelet P2Y12 receptor that irreversibly inhibits platelet activation; it is an inactive prodrug that requires two stages of hepatic metabolism by cytochrome P_450_ enzymes to become an active drug. This drug is affected by the process of absorption and metabolism, and the use of some medications can also affect its function [4, 5].

Ticagrelor is known as a potent oral inhibitor of the P2Y12 receptor and is a member of the cyclopentyl-triazolo-pyrimidines family and is one of the new antiplatelet drugs. Being an active metabolite, the drug inhibits platelet activity directly and reversibly and does not require hepatic metabolism; therefore, it has a faster onset of action than clopidogrel, and its antiplatelet effect is more robust and stable.

In patients with a STEMI diagnosis candidate for PPCI, it sounds logical to prescribe more potent antiplatelet drugs, such as ticagrelor or prasugrel. It has been stated in the recent STEMI guidelines that ticagrelor and prasugrel are preferred over clopidogrel. When these drugs are not available or are contraindicated, clopidogrel could be used as the P2Y12 inhibitor.

Previous studies have shown that ticagrelor is more effective than clopidogrel in preventing stent thrombosis and ischemic events among acute coronary syndrome (ACS) patients without increasing the risk of major bleeding [5–7]. The impact of initial thrombolysis in myocardial infarction (TIMI) flow on the outcome of STEMI has been investigated in some studies. It has been shown that higher initial TIMI flow is associated with lower in-hospital as well as 30-, 90-, and 180-day mortality. In addition, its desirable effects on stent thrombosis, cardiogenic shock, final infarct size, microvascular obstruction, reinfarction, ST-segment resolution, and final TIMI flow after angioplasty have been demonstrated [8, 9].

It is indicated in these studies that the initial TIMI flow of 2 and 3 was more beneficial in the clinical prognosis of patients compared to the initial TIMI flow of 0 or 1. On the other hand, in these studies, the initial TIMI flow was better in patients receiving ticagrelor than in patients taking clopidogrel [9, 10].

The impact of various P2Y12 inhibitors on STEMI outcomes is still a matter of debate. We evaluated the effect of ticagrelor on initial TIMI flow compared to clopidogrel in STEMI patients who are candidates for primary PCI (PPCI).

## 2. Methods

### 2.1. Study Design and Participants

This prospective cohort study was performed in Farshchian Heart Center, Hamadan University of Medical Sciences, Hamadan, Iran, from March 2018 to March 2019. Patients with acute STEMI in whom PPCI was indicated and consented to participate in the study were included. The diagnosis of acute STEMI was made according to the electrocardiograms prepared at admission to the emergency department and the necessary criteria for STEMI confirmation by experienced cardiologists. After STEMI was confirmed, patients were given a loading dose of clopidogrel (600 mg) or ticagrelor (180 mg) based on the discretion of the interventional cardiologist in charge of PPCI and prepared for emergency angiography and angioplasty. A loading dose of 325 milligrams of aspirin was prescribed for all aspirin-naive patients. Patients with a coronary artery bypass graft surgery history or ambiguous culprit vessels were excluded from the study.

### 2.2. Data Collection

Data regarding the baseline demographics comprising age, sex, history of hypertension (HTN), diabetes mellitus (DM), dyslipidemia (DLP), and current cigarette smoking were collected. Baseline angiographic variables included pain-to-door time, pain-to-wire time, culprit vessel, coronary dominancy, initial TIMI flow, final TIMI flow, and TIMI thrombus grading. The collected information about the antiplatelet drug prescribed before angiography was extracted from the patients' medical records, which were classified into two groups: those who received ticagrelor and those who received clopidogrel.

### 2.3. Outcomes

The primary outcome was the initial TIMI flow grade before performing PPCI. The secondary outcomes were the final TIMI flow grade, measured after PPCI, all-cause death, major adverse cardiovascular event (MACE, defined as a composite of all-cause death, myocardial infarction (MI), or stroke), and bleeding events defined according to the International Society on Thrombosis and Haemostasis (ISTH) criteria [11]. The relevant recorded angiography and angioplasty films were stored in the angiography unit system, and an independent interventional cardiologist, unaware of the patients' medical histories, determined the findings such as the culprit vessel, dominant coronary system, vessel score, TIMI thrombus grade, initial TIMI flow, and final TIMI flow.

The TIMI flow grade is a semiquantitative method for estimating epicardial coronary perfusion during angiography of infarct-related arteries. The TIMI grade flow ranges from 0 to 3, in which

  Grade 0 (no perfusion): there is no antegrade flow beyond the occlusion point of the infarct-related artery.
  Grade 1 (penetration without perfusion): the contrast media pass distal to the obstruction point but “hang up” and fail to opacify the entire infarct-related artery distal to occlusion for the duration of the filming sequence.
  Grade 2 (partial perfusion): the contrast media pass across the occlusion point and opacify the entire infarct-related artery bed distal to the obstruction. The rate of entry and clearance of contrast media into the infarct-related artery distal to the obstruction is slower than that in the opposite coronary artery.
  Grade 3 (complete perfusion): antegrade flow into the bed distal to the occlusion of the infarct-related artery is as fast as the antegrade flow into the proximal bed, and contrast clearance from the involved bed is as prompt as that from an uninvolved bed in the same vessel or the opposite artery [12].

The TIMI thrombus grade was classified according to the presence and size of the thrombus from grade 0 to 5 (Supplementary Table S1) [13].

### 2.4. Ethical Consideration

Informed consent was obtained from all participants. This study was approved by the Ethics Review Board of the Hamadan University of Medical Sciences. In all steps of the study, we strictly adhered to the Declaration of Helsinki [14].

### 2.5. Statistical Analysis

The data normality was checked using the Kolmogorov–Smirnov test. Qualitative variables were displayed as numbers and percentages. Chi-squared and Mann–Whitney *U* tests were used to analyze the differences between the nominal and ordinal qualitative variables between the two groups. Quantitative variables were displayed as the mean (standard deviation (SD)) or median (interquartile range (IQR)) and were analyzed using the Mann–Whitney *U* test or the independent *t*-test. Furthermore, an ordered logistic regression model was created to analyze the predictors of the primary outcome, and the results were reported as the odds ratio (OR) and 95% confidence interval (CI). The statistical significance level was considered less than 0.05. Data were analyzed using R version 4.2.1.

## 3. Results

### 3.1. Baseline Characteristics

From March 2018 to March 2019, 160 patients were included in the study, of whom 80 received clopidogrel and 80 received ticagrelor. No significant difference was observed between the treatment groups in terms of demographic characteristics and classical coronary artery disease risk factors (Table 1).

There was no significant difference between the groups regarding the mean (SD) pain-to-door time (154.18 (44.84) mins vs. 151.68 (42.31) mins, *p* value = 0.71) and pain-to-wire time (244.62 (44.57) mins vs. 240.71 (44.6) mins, *p* value = 0.58). A significant difference was observed between patients receiving ticagrelor and clopidogrel in terms of the culprit vessels (*p* value = 0.027). However, no significant difference was observed between the ticagrelor and clopidogrel groups in terms of the TIMI thrombosis grade (*p* value = 0.364), a marker of thrombus burden among STEMI patients, and dominant coronary system (*p* value = 0.056) (Table 1).

### 3.2. Primary Outcome

**Figure 1:**
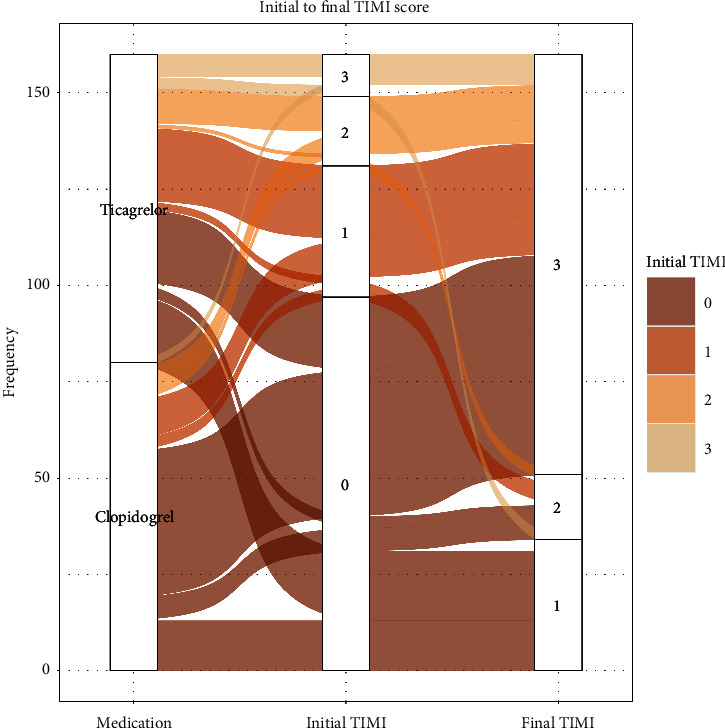
Graphical representation of the TIMI score based on the treatment groups and the trend of the TIMI score change from initial to final sessions. TIMI: thrombolysis in myocardial infarction.

A statistically significant difference was shown between the groups receiving ticagrelor and clopidogrel in terms of the initial TIMI flow grade (*p* value = 0.022 and *p* value = 0.005, yielded from Chi-squared and Mann–Whitney *U* tests, respectively). In the post hoc Chi-squared analysis, only the TIMI flow grade 0 showed a significant difference (ticagrelor: 40 (50%) vs. clopidogrel: 59 (71.2%), *p* value = 0.047). Overall, the difference between the two treatment groups in terms of the initial TIMI flow grade was statistically significant in favor of ticagrelor. No significant statistical difference was observed between ticagrelor and clopidogrel treatment groups in terms of the final TIMI flow grade (after angioplasty) (Table 2). The plot in Figure 1 visualizes the proportion of the initial TIMI score based on the treatment group and the trend of TIMI score change from initial to final angiograms.

An ordered logistic regression model was built to calculate the predictors of the primary outcome, the initial TIMI flow score. The logistic model was adjusted for age, sex, hypertension, diabetes mellitus, dyslipidemia, smoking, and treatment group (ticagrelor or clopidogrel). The results showed that ticagrelor was associated with a significantly higher TIMI flow grade compared to the clopidogrel group (adjusted OR: 2.90 (95% CI: 1.51–5.72), *p* value = 0.001). The results are given in Table 3.

### 3.3. Secondary Outcomes

Two (2.5%) all-cause deaths happened in the ticagrelor group and 3 (3.75%) deaths happened in the clopidogrel group and the difference was statistically not significant (*p* value = 0.65, yielded from Chi-squared test). No MI or stroke occurred in either group; thus, MACE happened in 2 (2.5%) and 3 (3.75%) patients in the ticagrelor and clopidogrel groups, respectively (no significant difference between the groups with a *p* value = 0.65, yielded from Chi-squared test). Two (2.5%) minor gastrointestinal bleeding events occurred in the ticagrelor group and no bleeding occurred in the clopidogrel group, showing no significant difference between the groups (*p* value = 0.16, yielded from Chi-squared test).

An ordered logistic regression model was built to calculate the predictors the final TIMI score. The model was adjusted for age, sex, hypertension, diabetes mellitus, dyslipidemia, smoking, initial TIMI score, and treatment group (ticagrelor or clopidogrel). None of the covariates were significantly associated with the final TIMI flow score. The results are represented in the Supplementary Table S2.

## 4. Discussion

This prospective study aimed to compare the initial TIMI flow in STEMI patients referred for PPCI who were treated with either ticagrelor or clopidogrel. Patients in the ticagrelor and clopidogrel groups had no significant difference in age, sex, or TIMI thrombus grade. The initial TIMI flow grade of the culprit artery was significantly higher in the ticagrelor group compared with that of the clopidogrel group. Furthermore, ticagrelor, compared to clopidogrel, significantly increased the odds of TIMI flow score improvement. However, there was no significant difference between the treatment groups in terms of the final TIMI flow grade.

PPCI is the standard and preferred treatment in STEMI with acceptable efficacy compared to other treatment options [15]. In a clinical trial conducted in 2019 by Cao et al. [16], the short-term outcomes of the STEMI patient candidates for PPCI who received ticagrelor or clopidogrel were compared. There was no significant difference between the two groups regarding TIMI flow grade zero, grade I, and grade II; However, grade III TIMI flow was more frequent in the ticagrelor group than in the clopidogrel group (*p*=0.016).

In an analysis of the Bremen STEMI registry conducted by Schmucker et al. in 2019 [17], the efficacy and safety of ticagrelor compared to clopidogrel in elderly STEMI patients who underwent PPCI were evaluated. There were 7466 STEMI patients, of whom 1087 patients over 75 were selected. It was observed that the initial TIMI flow of zero in the clopidogrel group was significantly higher than the ticagrelor group (*p*=0.04), and there was no significant difference between the two groups in terms of final TIMI flow, which are consistent with the results of our study.

In a systematic review conducted in 2019 to compare ticagrelor with clopidogrel in managing patients with acute myocardial infarction, the results of studies published in databases over the last ten years have shown that ticagrelor reduces the size of infarction and mortality compared to clopidogrel. However, the results based on angiographic studies did not indicate a significant difference between the two treatment methods [18].

In a study by Dai et al. [1] in 2017 on 4162 STEMI patients treated by PPCI, the results showed that the initial TIMI flow of the ticagrelor group was far superior to the clopidogrel group without significant difference in hemorrhagic events. Despite the smaller volume of our study, the results were consistent with that of Dai et al. [1].

Zhu et al. [19] in 2015 in China and Winter et al. [20] in 2014 in Chile also showed that the ticagrelor loading dose, in the early stages of acute myocardial infarction, inhibits platelet activity more effectively than clopidogrel, resulting in a better initial TIMI flow; however, the final TIMI flow did not differ much between the two groups, in line with the results of our study.

Studies have shown that better initial TIMI flow in STEMI patients is associated with a smaller size of infarction and microvascular occlusion [3]. It has been hypothesized that an initial TIMI flow of 2 or 3 compared to 0 or 1 is associated with shorter ischemia duration and a significant reduction in myocardial damage. Infarct size and microvascular obstruction could independently predict adverse left ventricular remodeling and major adverse cardiovascular events in STEMI patients. Assuming similar time delays from symptom onset to PPCI in both groups, patients with initial TIMI of 2 and 3 probably have shorter ischemic times compared to those with initial TIMI of 0 and 1. Another possible benefit is that the visible trajectory of the culprit vessel with TIMI 2 or 3 will lead to more straightforward wiring and possibly faster reperfusion.

Patients with higher initial TIMI flow before angioplasty will be more likely to have a higher TIMI flow after angioplasty. They will also have lower in-hospital as well as 30-day and 180-day mortality, stent thrombosis, reinfarction, and cardiogenic shock [6]. Other studies showed that despite having a significant effect on reducing cardiovascular mortality, myocardial infarction, and stent thrombosis compared to clopidogrel, ticagrelor administration is not associated with higher rates of cerebrovascular accidents (CVA) and bleeding risk [21, 22].

Ticagrelor has improved platelet inhibition in the early hours as well as maintenance treatment of STEMI patients [5]; hence, prehospital administration of ticagrelor has been recommended for patient candidates for PPCI [8].

Pretreatment with P2Y12 inhibitors was supported by strong recommendation at the time of our study (2018-2019), according to the 2017 European Society of Cardiology (ESC) guideline for the management of STEMI (class I and level of evidence A) [23]. On this basis, our patients were pretreated with ticagrelor or clopidogrel, and we did not compare them in this regard. In the recently published ESC guideline for the management of ACS, the pretreatment with P2Y12 inhibitors in STEMI is of a class IIb indication (weak recommendation), while in non-ST segment elevation ACS, routine pretreatment with P2Y12 inhibitors in patients whose coronary anatomy is not known and an early invasive strategy is planned and contraindicated (class III recommendation) [24].

The strengths of the present study are the prospective cohort design and comparison of initial TIMI flow among subjects receiving clopidogrel or ticagrelor in a relatively sufficient sample size. The limitations of this study are the lack of randomization and possibly the effect of confounding variables on the study results.

## 5. Conclusion

In STEMI patients who were candidates for PPCI, administering the antiplatelet drug ticagrelor on top of aspirin before angiography was associated with better initial TIMI flow grade and higher probability of improvement in TIMI flow compared to clopidogrel and aspirin.

## Figures and Tables

**Table 1:** Baseline demographic and angiographic characteristics.

Variable^1^	Ticagrelor (*n* = 80)	Clopidogrel (*n* = 80)	*P* ^2^	*P* ^3^	*P* ^4^
Age (years)	59.46 ± 13.11	61.34 ± 11.08	0.330^5^	—	—
Female, sex	22 (27.5)	15 (19.0)	0.204	—	—
Hypertension	30 (37.5)	34 (42.5)	0.519	—	—
Diabetes mellitus	22 (27.5)	16 (20.0)	0.265	—	—
Dyslipidemia	11(13.8)	12 (15.0)	0.822	—	—
Smoking	29 (36.3)	38 (47.5)	0.149	—	—
Pain-to-door time (minute)	154.18 (44.84)	151.68 (42.31)	0.71^5^		
Pain-to-wire time (minute)	244.62 (44.57)	240.71 (44.6)	0.58^5^		
*Culprit vessel*					
LAD	47 (58.8)	38 (47.5)	0.027^*∗*^	1.000	—
LCX	9 (11.3)	14 (17.5)		0.923	
RCA	24 (30.0)	28 (35.0)		1.000	
*Dominance*					
Right	67 (83.8)	54 (67.5)	0.056	0.100	—
Left	8 (10.0)	16 (20.0)		0.459	
Codominant	5 (6.2)	10 (12.5)		1.000	
*TIMI thrombus grade*					
0	2 (2.5)	0	0.364	1.000	0.110
1	5 (6.3)	1 (1.3)		1.000	
2	4 (5.0)	3 (3.8)		1.000	
3	7 (8.8)	7 (8.8)		1.000	
4	11 (13.8)	10 (12.5)		1.000	
5	51 (63.7)	59 (73.8)		1.000	

LAD, left anterior descending artery; LCX, left circumflex artery; *P*, *p* value; RCA, right coronary artery; TIMI: thrombolysis in myocardial infarction. ^*∗*^*p* value<0.05. ^1^Data are presented as numbers (%) or the mean ± standard deviation (SD). ^2^Chi-squared test. ^3^Chi-squared post hoc analysis with Bonferroni correction. ^4^Mann–Whitney *U* test. ^5^Independent *t* test.

**Table 2:** Initial and final TIMI flow grades of the culprit vessel in the treatment groups.

Grade^1^	Ticagrelor (*n* = 80)	Clopidogrel (*n* = 80)	*P* ^2^	*P* ^3^	*P* ^4^
*Initial TIMI flow*					
0	40 (50.0)	59 (71.2)	0.022^*∗*^	0.047^*∗*^	0.005^*∗*^
1	21 (26.2)	13 (16.2)		0.976	
2	10 (12.5)	8 (10.0)		1.000	
3	9 (12.9)	2 (2.5)		0.229	
*Final TIMI flow*					
1	21 (26.2)	13 (16.2)	0.179	0.732	0.414
2	6 (7.5)	11 (13.8)		1.000	
3	53 (66.3)	56 (70.0)		1.000	

^1^Data are presented as numbers (%). ^*∗*^*p* value <0.05. ^2^Chi-squared test. ^3^Chi-squared post hoc analysis with Bonferroni correction. ^4^Mann–Whitney *U* test.

**Table 3:** Ordered logistic regression of the predictors of the initial TIMI score.

Variable	Adjusted^†^ OR	95% CI	*p*
Age (years)	1.0118	0.98–1.04	0.395
Female, sex	1.66	0.71–3.85	0.240
Hypertension	1.14	0.56–2.33	0.713
Diabetes mellitus	0.77	0.35–1.65	0.509
Dyslipidemia	2.46	1.00–5.99	0.047
Smoking	1.32	0.62–2.78	0.464
Treatment group (Ticagrelor/clopidogrel)	2.90	1.51–5.72	0.001^*∗*^

CI, confidence interval; OR, odds ratio; *P*, *p* value. ^*∗*^*p* value <0.05. ^†^Model adjusted for age, sex, hypertension, diabetes mellitus, dyslipidemia, smoking, baseline TIMI flow score, and treatment group.

## Data Availability

The data used to support the findings of this study are available from the corresponding author upon reasonable request.

## Supplementary Materials

Supplementary Materials The supplementary file includes Tables S1 and S2, as mentioned in the text.
