# Supplementary material for: The Comparison of the Initial TIMI Flow Grade in Acute ST-Elevation Myocardial Infarction Patients Receiving Ticagrelor vs. Clopidogrel before Undergoing Primary Percutaneous Coronary Intervention: A Prospective Cohort Study
Source: Cardiol Res Pract. 2024 Feb 5;2024:6632656. doi: 10.1155/2024/6632656 (PMC10861275; doi:10.1155/2024/6632656)
Supplement: Supplementary Materials — The supplementary file includes Tables S1 and S2, as mentioned in the text. [file 6632656.f1.docx]

**Supplementary materials**

**Contents**

[**Table S1- TIMI thrombus grading.** 2](#_Toc157201581)

[**Table S2- Ordered logistic regression of the predictors of follow-up TIMI score.** 3](#_Toc157201582)

# **Table S1- TIMI thrombus grading.**

| Grade | Description |
| --- | --- |
| Grade 0 | No angiographic evidence of thrombus |
| Grade 1 | Angiographic features suggestive of thrombus (decreased contrast density, haziness of contrast, irregular lesion contour, a smooth convex meniscus at the site of a total occlusion, suggestive, but not firmly diagnostic of thrombus) |
| Grade 2 | Definite thrombus present in multiple angiographic projections (marked irregular lesion contour with a significant filling defect, the greatest dimension of thrombus is <1/2 vessel diameter) |
| Grade 3 | Definite thrombus appears in multiple angiographic views (greatest dimension from >1/2 to <2 vessel diameters) |
| Grade 4 | Definite large-size thrombus present (greatest dimension >2 vessel diameters) |
| Grade 5 | Definite complete thrombotic occlusion of a vessel (a convex margin that stains with contrast, persisting for several cardiac cycles) |

**Data adapted from Del Portillo et al. study** (1).

# **Table S2- Ordered logistic regression of the predictors of follow-up TIMI score.**

| Variable^1^ | Adjusted^†^ OR | 95% CI |  | *P* |
| --- | --- | --- | --- | --- |
| Age (year) | 0.99 | 0.97-1.03 |  | 0.001***^*^*** |
| Female Sex | 1.13 | 0.44-2.93 |  | 0.018***^*^*** |
| Hypertension | 0.57 | 0.26-1.24 |  | 0.334 |
| Diabetes mellitus | 0.57 | 0.23-1.27 |  | 0.991 |
| Dyslipidemia | 1.04 | 0.37-3.11 |  | 0.804 |
| Smoking | 1.29 | 0.56-2.96 |  | 0.156 |
| Baseline TIMI 1 flow | 5.70 | 2.12-18.40 |  | 0.156 |
| Baseline TIMI 2 flow | 5.24 | 1.49-25.21 |  | 0.944 |
| Baseline TIMI 3 flow | 2.07 | 0.51-10.80 |  | 0.549 |
| Treatment group (Ticagrelor/Clopidogrel) | 0.59 | 0.28-1.23 |  | 0.162 |

CI, confidence interval; OR, odds ratio; P, p-value; TIMI, thrombolysis in myocardial infarction.

***^*^*** P-value<0.05

^†^ Model adjusted for age, sex, hypertension, diabetes mellitus, dyslipidemia, smoking, initial TIMI flow score, treatment group.
